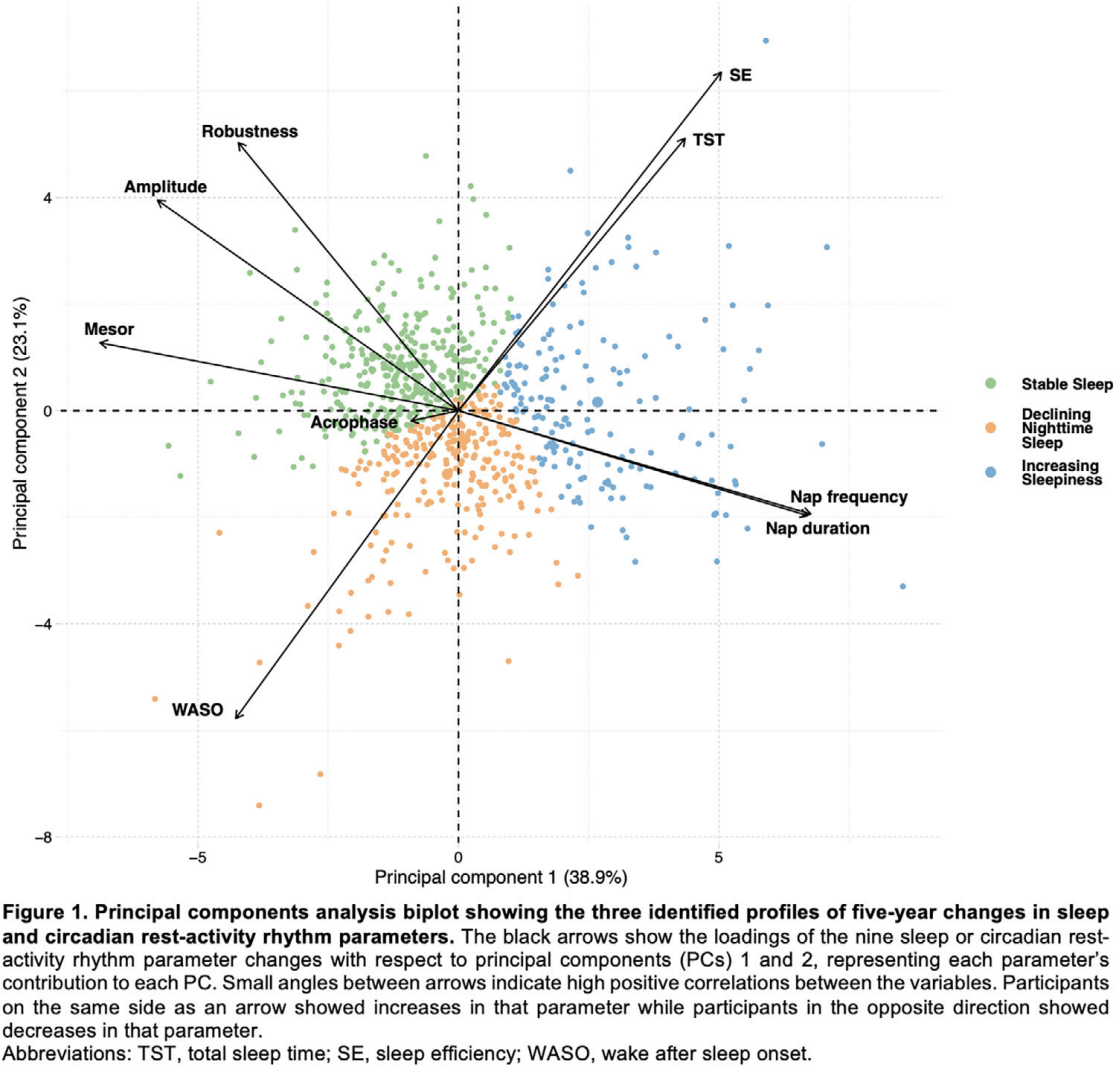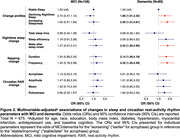# Five‐year changes in 24‐hour sleep‐wake activity and risk of mild cognitive impairment and dementia among older women

**DOI:** 10.1002/alz.094847

**Published:** 2025-01-09

**Authors:** Sasha Milton, Clémence Cavaillès, Sonia Ancoli‐Israel, Katie L Stone, Kristine Yaffe, Yue Leng

**Affiliations:** ^1^ University of California, San Francisco, San Francisco, CA USA; ^2^ University of California San Diego School of Medicine, San Diego, CA USA; ^3^ University of California, San Francisco and San Francisco VA Health Care System, San Francisco, CA USA

## Abstract

**Background:**

Sleep and circadian disruption have been associated with cognitive impairment in older adults. However, little is known about the longitudinal change in 24‐hour sleep‐wake activity in oldest old adults and how profiles of sleep‐wake changes may be associated with risk of mild cognitive impairment (MCI) and dementia.

**Method:**

We studied 769 women (mean age = 82.5 ± 2.9 years) without cognitive impairment at baseline who had nighttime sleep (sleep duration, sleep efficiency, and wake after sleep onset), napping (duration and frequency) and circadian rest‐activity rhythm (RARs; acrophase, amplitude, mesor, and robustness) parameters assessed using actigraphy at baseline (2002‐04) and follow‐up (2007‐08). The five‐year changes in all parameters were included in a hierarchical clustering on principal components analysis to identify patterns of sleep changes. We used multivariable multinomial logistic regression to evaluate the associations between these patterns and MCI and dementia (assessed at follow‐up).

**Result:**

During a mean follow‐up of 5.0 ± 0.6 years, 184 (24.0%) and 101 (13.1%) women developed MCI and dementia, respectively. We identified three sleep change profiles: 340 (44.2%) women with Stable Sleep (SS; stable sleep or slight improvement), 263 (34.2%) with Declining Nighttime Sleep (DNS; decreased nighttime sleep quality and duration and disrupted circadian RAR), and 166 (21.6%) with Increasing Sleepiness (IS; significant increases in both nighttime and daytime sleep duration and disrupted circadian RAR). After adjustment for age, race, education, body mass index, diabetes, hypertension, myocardial infarction, antidepressant use and baseline cognition, women with DNS [odds ratio, OR (95% confidence interval, CI) = 1.86 (1.01,3.42)] or IS [OR (95% CI) = 2.36 (1.21,4.61)] had approximately twice the risk of dementia compared to women with SS; patterns of sleep changes were not associated with MCI [OR (95% CI) = 0.72 (0.47,1.12) and 1.00 (0.61,1.64) for DNS and IS, respectively].

**Conclusion:**

Compared to those with stable sleep, older women with either declining nighttime sleep or increasing 24‐hour sleepiness over a five‐year period were twice as likely to develop dementia but not MCI. Decline in sleep health may be an important risk factor or marker of dementia in oldest old women.